# Cohort study: Neurological and cognitive-behavioral sequelae of acquired Zika virus infection among Nicaraguan children

**DOI:** 10.1038/s41390-024-03160-0

**Published:** 2024-07-02

**Authors:** Jill F. Lebov, Doré R. LaForett, Anna Gajewski, Erica N. Browne, José Victor Zambrana, Angel Balmaseda, Eva Harris, Stephen R. Hooper

**Affiliations:** 1 Social, Statistical, and Environmental Sciences, RTI International, Durham, NC USA; 2 FPG Child Development Institute, University of North Carolina at Chapel Hill, Chapel Hill, NC USA; 3 Sustainable Sciences Institute, Managua, Nicaragua; 4 Department of Epidemiology, School of Public Health, University of Michigan, Ann Arbor, MI USA; 5 Laboratorio Nacional de Virología, Centro Nacional de Diagnósis y Referencia, Ministerio de Salud, Managua, Nicaragua; 6 Division of Infectious Diseases and Vaccinology, School of Public Health, University of California, Berkeley, Berkeley, CA USA; 7 Department of Health Sciences, School of Medicine, University of North Carolina-Chapel Hill, Chapel Hill, NC USA

## Abstract

**Background:**

ZIKV has neuroinvasive properties, and in utero exposure can cause birth defects, but little is known about the neurological and neurocognitive impacts of acquired ZIKV infection, particularly in children.

**Methods:**

We assessed neurological symptoms frequency among ZIKV-infected children within one year after ZIKV infection. Three to 5 years post-infection, these children and a matched group of uninfected children were assessed via questionnaires, neurological exams, and neuropsychological testing to evaluate the association between prior ZIKV infection and subsequent neurological symptoms, and cognitive-behavioral function.

**Results:**

Among 194 ZIKV-infected children, 3 reported asthenia, 4 reported neck pain, and 10 reported back pain within one year post-infection. At follow-up, clinician-observed cranial nerve abnormalities were significantly more common among ZIKV-infected vs. uninfected children (16 vs. 3; *p* < 0.01), with vestibulocochlear nerve abnormalities observed most frequently. While ZIKV-infected children scored better than uninfected on cognitive measures, this difference was not clinically meaningful.

**Conclusions:**

Neurological signs, including paresthesia and cranial nerve abnormalities, were observed among ZIKV-infected participants in our study. However, we did not observe a meaningful link between acquired ZIKV infection and subsequent neurological, cognitive, or behavioral outcomes in a representative sample. An exception may be hearing impairment and loss, which should be explored further in future studies.

**Impact:**

- Neurological symptoms, though rare, were observed and reported more frequently among ZIKV-infected vs. uninfected children. These included: asthenia, neck pain, back pain, paresthesia, and cranial nerve abnormalities.
- Neurocognitive and behavioral test scores were similar among ZIKV-infected and uninfected children.
- Our study suggests that ZIKV-infected children should be monitored for neurological symptoms and cranial neuropathy to better understand the full burden of acquired ZIKV infection among children.

## Introduction

The Zika epidemic in 2015–2016 impacted millions of families in the Americas.^1^ Once initial reports emerged of congenital defects associated with in utero ZIKV exposure,^2^ the focus of ZIKV research shifted from acquired ZIKV infection to understanding the burden and mechanisms of maternal-fetal transmission and associated adverse outcomes.^3^ In contrast to congenital ZIKV infection, acquired ZIKV infection appears to have mostly mild symptoms. However, published Zika case reports and case series have described the presence of ZIKV RNA in cerebrospinal fluid of postnatally exposed children as well as acute and persistent neurological manifestations and neuropsychiatric symptoms in adolescents and adults.^4–9^ In vivo studies of ZIKV-associated neuropathology showed that ZIKV infection can cause chronic neuroinflammation, neuronal cell death, and brain function alterations in postnatally exposed mice and non-human primates.^10–12^ The neurotropic properties of ZIKV raise concern about neurodevelopmental consequences associated with postnatal ZIKV infection. Whether ZIKV-associated neurological sequelae are temporary and whether neuroinvasiveness of ZIKV is associated with subsequent cognitive or behavioral deficits in otherwise healthy children is unknown.

Epidemiological studies of the neurological health consequences of acquired ZIKV infection have been limited. Several studies attempted to track neurological outcomes in children but were unable to determine whether the frequency of observed problems was greater than that in the general population due to a lack of comparison populations.^13,14^ We conducted a pilot study of neurological, cognitive, and behavioral outcomes among ZIKV-infected and uninfected children in León, Nicaragua.^15^ Though our sample size was not large enough to draw conclusions, we did observe a small percentage of ZIKV-infected children with abnormal responses to basic neurological tests. In addition, we found our battery of measurement tools to be feasible for use in similar populations. Building on the pilot study, we designed a larger study in Managua, the capital city of Nicaragua, to evaluate the presence and persistence of neurological symptoms in children with postnatally acquired Zika infection. We also investigated associations between acquired ZIKV infection and long-term neurologic impairment, neuropsychological deficits, social-behavioral functioning, and auditory sequelae.

## Methods

### Study population

The source population for this study was children enrolled in the Pediatric Dengue Cohort Study (PDCS). The PDCS is a community-based cohort study of dengue among children aged 2-17 years old in Managua, Nicaragua. The study has been continuously running since 2004; ZIKV testing was added to the cohort testing protocol in July 2015. At the first sign of illness or fever, children participating in the PDCS are brought by their parents/guardians to the primary public health clinic (Socrates Flores Vivas) for a study visit. Details about the PDCS have been described elsewhere.^16,17^

### Symptomatic and asymptomatic ZIKV infection

An acute blood sample was collected from all children with arbovirus disease-like symptoms or undifferentiated febrile illness and was tested for dengue virus (DENV), chikungunya virus (CHIKV), and ZIKV using multiplex real-time reverse transcriptase PCR (rRT-PCR).^18^ A convalescent sample was collected during days 14–21; paired serology was performed using the acute and convalescent samples to capture any ZIKV-positive children who were missed using the rRT-PCR test. ZIKV symptomatic infections were defined with ZIKV rRT-PCR, ZIKV-specific IgM ELISA or ZIKV inhibition enzyme-linked immunosorbent assay (iELISA) seroconversion, a 4-fold or greater increase in ZIKV-specific total antibodies captured by ZIKV iELISA, and/or positivity by ZIKV NS1 Blockade-of-Binding (BOB) ELISA^19^ without evidence of DENV seroconversion, DENV iELISA ≥4-fold change, or DENV rRT-PCR positivity.^20,21^

Additionally, all cohort children provide annual blood samples in March that are tested for serological evidence of DENV, CHIKV, and ZIKV infection using the ZIKV iELISA and ZIKV NS1 BOB ELISA assay, DENV iELISA, and CHIKV iELISA.^19,20,22,23^ Children were considered ZIKV-infected if they had a symptomatic ZIKV infection or if they seroconverted from one annual sample to the next, without evidence of DENV seroconversion, in 2016, 2017, and 2018.^24^

### Baseline symptoms data

Each time PDCS participants present to the clinic with symptoms of febrile illness, they are assessed for >80 signs and symptoms, including asthenia, seizures, loss of consciousness, fatigue, back pain, neck pain, and neck rigidity.^16^ Children with suspected ZIKV infection were also administered a questionnaire at both the initial and convalescent visits to ascertain information about common Zika symptoms (e.g., rash, fever, muscle, joint pain), neurological symptoms (e.g., paralysis, paresthesias, persistent headaches, muscle weakness, seizures, fainting/blackouts, lethargy/fatigue, back pain), and respiratory and gastrointestinal symptoms. In this study, we included questionnaire data from the initial and convalescent Zika visits and data from all febrile illness visits that occurred within one year after each participant’s ZIKV infection was identified.^21^

### Follow-up study

A follow-up study was conducted from November 2019 to June 2021. Children from the PDCS cohort who were previously identified as ZIKV-infected and uninfected (as defined above) were eligible for the follow-up study if they did not have a known pre-existing neurological or psychological condition or head trauma and if they were aged 5-16 years old at the time of follow-up study screening. We chose this age range to ensure that ZIKV-infected children were not exposed to ZIKV *in utero*. Children with inconsistent or missing ZIKV test results were excluded from recruitment.

All eligible confirmed ZIKV-infected children and a random sample of ZIKV-uninfected children, selected to maintain the same distribution of age and sex as the ZIKV-infected participants, were recruited to evaluate potential long-term sequelae 3–5 years after ZIKV exposure. Two study visits were conducted in a dedicated clinic space in Managua. In one visit, a medical exam and medical history questionnaire were administered. In the other visit, neuropsychological testing and risk factor questionnaires were administered. Participants were randomized to receive the medical visit or the neuropsychological visit first to help reduce missingness in a certain set of measurements due to loss-to-follow-up between the first and second visits. During the COVID-19 pandemic when in-person study visits were not possible, questionnaires were administered over the phone to maintain study continuity.

As part of the questionnaires, we asked participants’ parents about changes since the Zika epidemic in their child’s level of fatigue, motor functioning, cognitive abilities (i.e., ability to think or concentrate), and changes in hearing or vision. We also asked about head injuries since enrollment to ensure that the participants did not sustain a recent head injury that could result in neurological symptoms or cognitive impact. We were not able to directly assess lead exposure; instead, we used family work in a battery shop as a potential proxy for lead exposure.^25^ Adolescents were asked about their exposure to neurotoxic pesticides (i.e., propoxur, cypermethrin, temephos, pyrethroids, imiprothrin, chlorpyrifos). We asked about family history of neurological (i.e., stroke, epilepsy, multiple sclerosis, or other brain disease) and psychological (i.e., anxiety, depression, schizophrenia, alcoholism, drug abuse, learning disabilities, attention deficit) problems. We used the PROMIS Family Relationship Measure Parent Proxy report (8a) to evaluate family relationships.

A medical examination, including a neurological assessment, was conducted by licensed study physicians. The exam included an evaluation of all body systems, a questionnaire about experience of neurological symptoms during the previous 6 months, and a basic assessment of muscle tone, strength, reflexes, and cranial nerves. A composite endpoint variable was developed in the analysis phase to combine observations of cranial nerve abnormality or abnormal muscle tone, reflexes, or strength not associated with a known medical condition or accident, or new diagnosis of a neurological disorder. Distortion product otoacoustic emissions testing (frequency range: 1.0-4.9 kHz) was conducted for hearing screening. Regardless of the outcome of otoacoustic emissions testing, cranial nerve 8 testing was conducted by rubbing the fingers by one ear while occluding the other, then repeating for the other ear. Any asymmetry in response or hearing was noted. Further evaluation using the Rinne and Weber tests was conducted if hearing loss was suspected. Neuropsychological tests were conducted in a quiet private room by local study psychologists trained by a licensed psychologist from the United States on the administration of the Batería IV Woodcock-Muñoz (Batería IV™) Pruebas de Habilidades Cognitivas and the Test of Nonverbal Intelligence 4 (TONI-4). The Batería IV was designed to assess the cognitive abilities of Spanish-speaking children and adults, and the TONI-4 is a language-free test that uses figures void of any cultural references or symbology to assess intelligence, aptitude, abstract reasoning, and problem-solving. The same psychologist also trained study nurses on the administration of the Child Behavior Checklist (CBCL) to a parent or caregiver. These instruments had been piloted in a smaller study in León, Nicaragua,^15^ and the principal investigator from the León study team met with the Managua study team to discuss lessons learned from the pilot study.

Clinical staff were blinded to ZIKV infection history at the time of assessment. The study protocol was reviewed and approved by the Institutional Review Boards (IRBs) of the Ministry of Health of Nicaragua and RTI International. Prior to initiating study activities, parents provided consent, and children aged 6–16 years old provided assent. If the parent provided consent but the child did not assent, the child was not enrolled in the study.

### Statistical methods

Demographic characteristics, possible confounders, and outcomes were summarized by ZIKV exposure; categorical measures were summarized with frequencies and percentages, and continuous data were summarized by presenting mean, standard deviation, median, and interquartile range (IQR). For binary outcomes, Chi-squared tests (or Fisher’s exact test when cell size *n* < 5) were used in univariate analyses to compare outcomes by ZIKV exposure.

Two-sample Wilcoxon rank-sum (Mann-Whitney) test was used to compare continuous outcomes by ZIKV infection. Neuropsychological test scores of the Batería IV Woodcock-Muñoz (Batería IV) < 40 are not given an exact score; therefore, scores noted as <40 were analyzed as a score of 39. In multivariable-adjusted models, factors that were univariably associated with the outcome and ZIKV infection with *p* < 0.10 were included in the models as covariates. Factors thought to be relevant based on scientific literature were also considered for inclusion as covariates. The primary quantitative outcome measures of interest, neurocognitive functioning scores, were compared by ZIKV infection using an ANCOVA model. All analyses were conducted using Stata 16.1 (StataCorp, College Town, TX).

## Results

### Study population

**Fig. 1:**
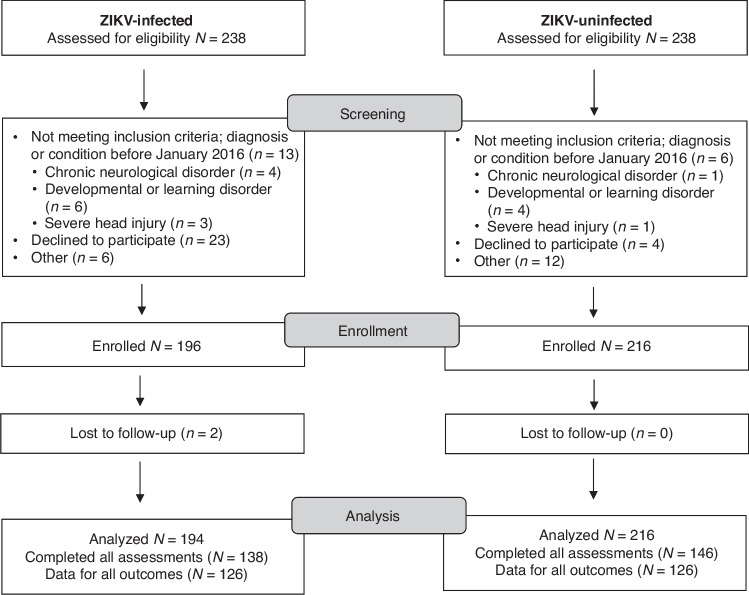
ZeN study screening, enrollment, and analysis population. 238 children were assessed for eligibility in the ZIKV-infected and uninfected groups. After exclusions and loss to follow up, the data for 194 ZIKV-infected and 216 ZIKV-uninfected participants were included in statistical analyses.

From January 1, 2016, to December 31, 2016, 3,460 children were actively followed via the PDCS. Of these, there were 560 Zika cases, including 374 RT-PCR positive cases. Between 2016 and the start of this study in 2019, 292 participants were still active in the PDCS when recruitment began. Of these, 43 could not be reached for recruitment and 11 children had aged out of the maximum age criteria by the end of the recruitment period. This left 238 previously ZIKV-infected children for the study (henceforth referred to as ‘ZIKV-infected’ or ‘infected’). As controls, 238 ZIKV-uninfected children of the same age and sex distribution were randomly selected from the PDCS cohort and contacted to assess eligibility criteria. In total, 194 ZIKV-infected and 216 uninfected children were enrolled (Fig. 1). Infected and uninfected children had similar distributions by sex and age (by design), as well as comparable parental employment, family history of neurological disease, and school type (Table 1). No participants reported a head injury since enrollment (3 responses were missing). ZIKV-infected children reported weaker family relationships, were more likely to be exposed to neurotoxic pesticides in the last 6 months, and were more likely to have a biological parent with a history of a psychological condition (Table 1). Twenty-seven percent of parents in both groups considered their children to learn slower than others. Grade repetition, sleep issues, and anxiety were observed in a quarter to one-half of participants in each group. Our proxy indicator of potential lead exposure showed limited battery shop exposure in both groups. Very little alcohol and drug use was reported among 13–17-year-olds, with no variability by ZIKV status (data not shown). Four ZIKV-infected children had abnormal otoacoustic emissions test results; all uninfected children had normal results.

**Table 1:** Characteristics of ZIKV-infected and ZIKV-uninfected children in Managua, Nicaragua.

	ZIKV-infected		ZIKV-uninfected		Total		
	*N*	(%)	*N*	(%)	*N*	(%)	*p*-value
Total enrolled	194		216		410		
Age—mean, median (IQR)	10.6, 11	(9–13)	10.8, 11	(9–13)	10.7, 11	(9–13)	0.70
Female	106	(55)	119	(55)	225	(55)	0.93
**Completed family and household exposure questionnaire**	187		204		390		
Parent/guardian employment							0.37
None	22	(12)	34	(17)	56	(14)	
One	110	(59)	117	(58)	227	(58)	
Two	54	(29)	53	(26)	107	(27)	
Family relationship, *t*-score—*mean, median (IQR)*^*a*^	56.1, 56.1	(51.8–63.1)	57.5, 63.1	(54.3–63.1)	56.8, 57.6	(52.0–63.1)	0.01
Those with a family relationship t-score equal to the max (63.1)	76	(41)	113	(55)	189	(48)	0.005
Proxy for potential lead exposure^b^	5	(3)	2	(1)	7	(2)	0.27
High neurotoxic pesticide exposure, past 6 months^c^	33	(18)	19	(10)	52	(14)	0.02
*Family relative*							
History of neurological disease^d^	23	(12)	38	(19)	61	(16)	0.21
History of psychological condition	62	(33)	59	(29)	121	(31)	0.60
*Biological parent*							
History of neurological disease^d^	8	(5)	9	(5)	17	(5)	1.00
History of psychological condition	41	(27)	30	(19)	71	(23)	0.08
Missing, parent not at study visit	35		38		72		
**Completed child characteristics questionnaire**	190		205		395		
School attendance							0.27
Private	55	(29)	45	(22)	100	(25)	
Public	129	(68)	152	(74)	281	(71)	
Not attending school	6	(3)	8	(4)	14	(4)	
Attends program for children with learning disabilities	1	(1)	0	(0)	1	(0)	0.30
Has repeated a grade in school	46	(24)	63	(31)	109	(28)	0.15
Parent/guardian thinks child learns slower than others	52	(27)	55	(27)	107	(27)	0.93
**Completed physical exam**	173		178		351		
Body mass index							0.10
Underweight (<5th percentile)	6	(4)	7	(4)	13	(4)	
Healthy weight	103	(60)	120	(67)	223	(64)	
Overweight (85–97th percentile)	39	(23)	22	(12)	61	(17)	
Obese (>97th percentile)	25	(14)	29	(16)	54	(15)	
Clinical evidence of malnutrition	21	(12)	18	(10)	39	(11)	0.55
**Psychological covariates**							
Completed BEARS questionnaire	188		205		393		
Bedtime problems	31	(17)	43	(21)	74	(19)	0.26
Awakenings during the night	35	(19)	55	(27)	90	(23)	0.05
Bedtime after 10 pm	29	(16)	34	(17)	63	(16)	0.77
Snoring	45	(24)	49	(24)	94	(24)	0.99
Completed CDI-2:SR[S]	169		186		355		
Depression score—*mean, median (IQR)*	4.5, 4	(2–6)	4.5, 4	(2–7)	4.5, 4	(2–6)	0.80
Completed Spence	163		149		312		
Anxiety, *t*-score—*mean, median (IQR)*	38.6, 38	(26–49)	41.1, 38	(28–51)	39.9, 38	(27–51)	0.36
Elevated anxiety	63	(45)	86	(50)	149	(48)	0.44

^a^Higher scores indicate a stronger parent-child relationship.

^b^Someone in the family works in a battery shop.

^c^Daily or weekly use of propoxur, cypermethrin, temephos, pyrethroids, imiprothrin, and/or chlorpyrifos.

^d^Stroke, epilepsy, multiple sclerosis, and other brain diseases.

CDI-2: Children’s Depression Inventory, Second Edition: Self-Report Short version in children aged 7–17 years.

### Neurologic symptoms

In the baseline study, 3 ZIKV-infected participants reported asthenia, 10 reported back pain, and 4 reported neck pain (data not shown). There were no reported instances of seizure, loss of consciousness, lethargy, neck stiffness, muscle weakness, paresthesia, or paralysis.

In the follow-up study, the composite outcome of self-reported neurological symptoms (any symptom) in the prior 6 months was more frequently reported among the ZIKV-infected group (9%) compared to the uninfected group (6%); however, this difference was not statistically significant (*p* = 0.20) (Table 1). Paresthesia, the most reported symptom, was reported by 15 children (6% infected, 2% uninfected). The composite variable (any symptom) for clinically observed neurological symptoms was reported significantly more often (*p* = 0.005) in infected (11%) than uninfected (3%) children. This result was driven by differences in observed cranial nerve abnormalities (9% infected, 2% uninfected; *p* = 0.002).

Observed vestibulocochlear nerve (CN 8) abnormalities were more common among ZIKV-infected children (6% infected, 1% uninfected; *p* = 0.007) (Table 1). Clinician-observed optic and oculomotor cranial nerve (CN II and CN III) abnormalities included strabismus (*n* = 3 in ZIKV-infected vs. *n* = 1 in uninfected) and decreased visual acuity (*n* = 1 in ZIKV-infected vs. *n* = 0 in uninfected), though it is unclear if these conditions initiated prior to or after ZIKV exposure. No other cranial nerve abnormalities were observed. Abnormalities in muscle tone, strength, and reflexes were also absent in both groups (data not shown).

### Cognitive and social-behavioral function

Results of the cognitive and behavioral assessments are presented in Table 2. In models adjusted for repeated grade in school, pesticide exposure, family relationship score, and parent with a history of psychological condition, statistically significantly higher (better) scores were seen for the cognitive assessments for the ZIKV-infected compared to uninfected children. However, the average standardized scores of the ZIKV-infected group were within a 90% confidence interval for the standard error of measurement (i.e., about ±7 points), thus likely reflecting normal variation in the cognitive measures (i.e., a clinically insignificant difference) (Table 3). There were no statistically or clinically significant differences in the scores for the Internalized, Externalized, or Total Behavior scores of the behavioral assessment (CBCL). Additional adjustments for parent employment, attending private school, waking at night, and test administrator showed similar results for both the cognitive and social-behavioral outcomes (data not shown). Failed hearing screening was not associated with findings from the cognitive testing.

**Table 2:** Neurologic Symptoms in ZIKV-infected vs. ZIKV-uninfected in Managua, Nicaragua.

	ZIKV-infected		ZIKV-uninfected		Total		
	*N*	(%)	*N*	(%)	*N*	(%)	*p*-value
**Neurological symptoms in last 6 months, self-report**	177		196		373		
Any symptom	16	(9)	11	(6)	27	(7)	0.20
Seizures	1	(1)	3	(2)	4	(1)	0.63
Stiff neck	0	(0)	0	(0)	0	(0)	-
Muscle paralysis	0	(0)	0	(0)	0	(0)	-
Muscle weakness	1	(1)	4	(2)	5	(1)	0.21
Paresthesia	11	(6)	4	(2)	15	(4)	0.06
Neuropathic pain	3	(2)	1	(1)	4	(1)	0.35
**Hearing screening**	170		178		348		
Abnormal^a^	4	(2)	0	(0)	4	(1)	0.06
**Clinically observed neurological symptoms**	171		176		347		
Any symptom	18	(11)	5	(3)	23	(7)	0.005
Cranial nerve abnormality	16	(9)	3	(2)	19	(6)	0.002
II: Vision	2	(1)	0	(0)	2	(1)	0.24
III, IV, VI: Eye movements	4	(2)	1	(1)	5	(1)	0.21
VII: Facial movement	1	(1)	0	(0)	1	(0)	0.49
VIII: Hearing	11	(6)	2	(1)	13	(4)	0.007
Other abnormalities not related to medical conditions or accident	9	(5)	4	(2)	13	(4)	0.17

^a^Failed test in at least one ear.

**Table 3:** Cognitive and social-behavioral functioning in ZIKV-infected vs. ZIKV-uninfected.

	**ZIKV-infected**		**ZIKV-uninfected**		**Total**		**Adj mean diff**	**95% CI**	***p*-value**
	**Mean (SD)**	**Median (IQR)**	**Mean (SD)**	**Median (IQR)**	**Mean (SD)**	**Median (IQR)**			
**Completed cognitive assessments**—***N***	179		189		368				
Batería Woodcock-Muñoz IV^a^									
Short-term working memory, Composite^b^	82.1 (16.8)	85 (73–95)	75.6 (18.3)	77.5 (63–88)	78.8 (17.8)	81 (67–92)	6.2	(2.6, 9.7)	0.001
Verbal attention	86.1 (16.0)	87 (78–98)	81.0 (18.1)	84 (71–94)	83.5 (17.3)	86 (75–96)	5.2	(1.7, 8.8)	0.004
Visualization	80.2 (14.6)	81 (71–92)	80.2 (13.8)	81 (71–90)	80.2 (14.2)	81 (71–91)	–0.7	(–3.7, 2.3)	0.66
Pair cancellation	82.3 (15.1)	83 (75–92)	79.3 (13.3)	80 (71–88)	81.0 (14.3)	81 (72–90)	3.5	(0.5, 6.4)	0.02
Oral vocabulary^c^	78.8 (20.5)	80.5 (65–92)	75.5 (16.7)	77 (66–86)	77.1 (18.7)	79 (65–90)	3.1	(–0.7, 6.9)	0.11
Number facility, composite^d^	77.8 (17.8)	82 (67–90)	71.3 (18.5)	73 (59–85)	74.5 (18.4)	77 (62–87)	5.5	(1.7, 9.3)	0.005
Number series	71.5 (18.0)	72 (58–86)	66.6 (17.9)	66 (53–82)	69.0 (18.1)	69 (54–83)	4.4	(0.7, 8.1)	0.02
Numbers reversed	83.4 (17.3)	87 (74–96)	77.9 (17.8)	78 (65–91)	80.6 (17.8)	82 (70–94)	5.7	(2.1, 9.4)	0.002
Number-pattern matching	80.0 (17.7)	83 (71–91)	74.6 (19.3)	77 (60–91)	77.2 (18.7)	81 (67–91)	4.5	(0.6, 8.4)	0.03
Test of Nonverbal Intelligence (TONI)^a^	87.4 (7.8)	88 (82–92)	84.6 (8.3)	84 (79–90)	85.9 (8.2)	85 (80–91)	2.6	(0.9, 4.2)	0.003
**Completed child behavior checklist (CBCL)**^**e**^—***N***	181		192		373				
Internalizing problems	61.2 (9.5)	63 (54–69)	58.2 (10.7)	58 (52–66)	59.7 (10.2)	61 (52–68)	1.7	(–0.4, 3.8)	0.11
Externalizing problems	57.6 (10.5)	58 (51–65)	59.4 (9.1)	60 (53–66)	58.5 (9.9)	59 (51–65)	1.1	(–0.9, 3.1)	0.29
Total problems	60.6 (8.7)	60 (54–68)	58.2 (10.1)	59 (51–65)	59.4 (9.5)	60 (53–67)	1.4	(–0.5, 3.3)	0.15

^a^The Batería IV and TONI-4 have standardized scores with a mean of 100 and an SD of 15. Higher scores indicate better cognitive function.

^b^Composite includes verbal attention and numbers reversed.

^c^*N* = 331 (ZIKV+ *n* = 160, ZIKV– *n* = 171).

^d^Composite includes numbers reversed and number-pattern matching.

^e^The CBCL scores are *t*-scores with a mean of 50 and SD of 10. Higher scores indicate a worse behavioral rating.

Adj mean diff: adjusted mean difference. Regression models adjusted for repeated grades in school, pesticide exposure, high family relationship score, and a parent with a history of psychological condition.

## Discussion

In the current study, we evaluated neurological, cognitive, and social-behavioral outcomes among a well-characterized population of children with and without acquired ZIKV infection to assess short-term sequelae and to understand if the occurrence of long-term sequelae was associated with prior ZIKV infection.

We found several instances of symptoms potentially related to neurologic complications among the 194 ZIKV-infected children: asthenia, neck pain, back pain, paresthesia, and cranial nerve abnormalities. Previous studies have reported a variety of central and peripheral nervous system manifestations among ZIKV-infected patients, the most prominent of which is Guillain-Barré Syndrome (GBS), but which also included myelitis, encephalitis, sensory polyneuropathy, and chronic inflammatory demyelinating polyneuropathy.^4,26–32^ Lannuzel et al.^13^ published a larger study of neurological outcomes associated with acquired ZIKV infection among children and adults in the French West Indies. Among 6 children included in the study, 2 had GBS, 2 had encephalitis-myelitis, 1 had a stroke, and 1 had a mixed disorder.^13^ While this study was important, as a case series it was unable to elucidate whether the prevalence of neurological outcomes among this population was greater than in the general population. Our study is unique because the cohort design allows us to compare long-term neurological sequelae in children with acquired ZIKV infection vs. uninfected children from the same source population. Further, the strain of ZIKV circulating in French Polynesia in 2013 was different than that in Nicaragua in 2016. Given that various strains infect and impact human brain cells differently, the results from Lannuzel’s study may not be generalizable to the Americas.^33^

To our knowledge, our study represents the largest pediatric cohort to date to evaluate neurological symptoms at the time of postnatally acquired infection and years later. Trained physicians, nurses, and psychologists administered standardized tests that were pre-tested in Nicaragua, with numerous data domains captured and intensive work to ensure data quality. Among study participants, we did not see any major differences in neurological signs and symptoms nor any differences in neurocognitive and social-behavioral outcomes associated with ZIKV infection. While there were more reported instances of paresthesia among ZIKV-infected children, this difference did not reach statistical significance, and paresthesia could be due to any number of unobserved conditions other than prior ZIKV infection.

Sensorineural hearing loss has been observed among children with congenital ZIKV syndrome.^34^ An adult with confirmed ZIKV infection in Bahia reported experiencing transient sensorineural hearing loss, but this was in the acute phase of the disease.^35^ Long-term hearing loss has been observed among adults with West Nile virus infection, another neuroinvasive flavivirus.^36^ In our follow-up study 3–5 years after the Zika epidemic, we observed abnormal hearing screening and cranial nerve test results more commonly among the ZIKV-infected children, but it is unclear if the hearing impairment began before or after ZIKV exposure. Future studies should examine this question to a greater degree.

The score means and IQRs in both exposure groups were below the standard mean of the cognitive and behavioral instruments. In addition, nearly half of the participants had elevated anxiety. Low cognitive scores, behavioral problems, and elevated anxiety among cohort participants may stem from the low-resource circumstances of participating families.

### Limitations

It is possible that instances of more severe neurological manifestations of ZIKV infection were missed in this study. The children in this study were evaluated around the time of infection in the local health clinic. Children with severe neurological conditions, such as cranial nerve palsies or encephalitis, may have gone directly to the local hospital instead of the primary health clinic. However, the intake form for PDCS children who come to the clinic includes a question about the reason for hospitalization within the last year. There were no neurological conditions among the reasons reported. Still, it is possible that a severe neurological outcome caused the child to drop out of the PDCS, in which case the outcome would not be present in our dataset. It is also possible that the PDCS enrollment age minimum of 2 may have precluded observation of major neurological impacts of postnatal exposure, as other studies have observed long-term neurodevelopmental impairment among humans and non-human primates that experience ZIKV infection during infancy.^37,38^

Cognitive scores were better among ZIKV-infected children compared to uninfected, although the difference was not clinically meaningful and was not explained by confounding by other measured risk factors. The results from this study may represent a lack of meaningful impact of ZIKV on cognition and behavior among pediatric populations that are already experiencing the effects of poverty and instability.

We aimed to conduct study visits within 3 years of the ZIKV epidemic, but the COVID-19 pandemic delayed assessments among many participants. Because of the long delay between baseline ZIKV testing and our follow-up study, other issues or conditions, including COVID-19, may have arisen that could have impacted participants’ cognitive and behavioral development and other health indicators. There are many factors common among study participants (e.g., poor nutrition, limited educational stimulation outside of school, exposure to civil unrest) that could have affected the children’s working memory, processing speed, abstract reasoning, and behavior. Though we attempted to measure and control for potential confounding factors, it is possible that a myriad of other negative stimuli may have obscured any observable impact of ZIKV on participants’ neuropsychological test scores and social-behavioral ratings.

Most of the children in our study had at least one DENV infection at some point in the past. While this may limit generalizability to populations without DENV exposure (e.g., travelers), most regions that experienced a ZIKV epidemic were also DENV-endemic areas.

## Conclusions

Neurological signs, including paresthesia and cranial nerve abnormalities, were observed among a few participants in our study. We did not observe a meaningful link between acquired ZIKV infection and subsequent neurological, cognitive, or social-behavioral outcomes in a representative sample. A possible exception may be hearing impairment and loss, which should be explored further in future studies. Although Guillain-Barré syndrome, encephalitis, myelitis, and other neurological outcomes are associated with ZIKV, they appear to be infrequent in children. ZIKV is still circulating and could potentially re-emerge in the coming decades. Given the neurotropic properties of ZIKV and other mosquito-borne viruses, we recommend the inclusion of clinically observed or self-reported neurological symptoms and cranial neuropathy in future syndromic surveillance and observational research efforts in geographic areas where these viruses typically circulate.

## Data Availability

The datasets generated during the current study are not publicly available due to funding limitations but are available from the corresponding author upon reasonable request.
